# Regulating the Configurational Entropy to Improve the Thermoelectric Properties of (GeTe)_1−*x*_(MnZnCdTe_3_)*_x_* Alloys

**DOI:** 10.3390/ma15196798

**Published:** 2022-09-30

**Authors:** Yilun Huang, Shizhen Zhi, Shengnan Zhang, Wenqing Yao, Weiqin Ao, Chaohua Zhang, Fusheng Liu, Junqin Li, Lipeng Hu

**Affiliations:** 1 College of Materials Science and Engineering, Shenzhen Key Laboratory of Special Functional Materials, Guangdong Research Center for Interfacial Engineering of Functional Materials, Guangdong Provincial Key Laboratory of Deep Earth Sciences and Geothermal Energy Exploitation and Utilization, Institute of Deep Earth Sciences and Green Energy, Shenzhen University, Shenzhen 518060, China; 2 Superconducting Materials Research Center, Northwest Institute for Nonferrous Metal Research, Xi’an 710016, China

**Keywords:** thermoelectric, GeTe, entropy engineering, phase transition, lattice distortion

## Abstract

In thermoelectrics, entropy engineering as an emerging paradigm-shifting strategy can simultaneously enhance the crystal symmetry, increase the solubility limit of specific elements, and reduce the lattice thermal conductivity. However, the severe lattice distortion in high-entropy materials blocks the carrier transport and hence results in an extremely low carrier mobility. Herein, the design principle for selecting alloying species is introduced as an effective strategy to compensate for the deterioration of carrier mobility in GeTe-based alloys. It demonstrates that high configurational entropy via progressive MnZnCdTe_3_ and Sb co-alloying can promote the rhombohedral-cubic phase transition temperature toward room temperature, which thus contributes to the enhanced density-of-states effective mass. Combined with the reduced carrier concentration via the suppressed Ge vacancies by high-entropy effect and Sb donor doping, a large Seebeck coefficient is attained. Meanwhile, the severe lattice distortions and micron-sized Zn_0.6_Cd_0.4_Te precipitations restrain the lattice thermal conductivity approaching to the theoretical minimum value. Finally, the maximum *zT* of Ge_0.82_Sb_0.08_Te_0.90_(MnZnCdTe_3_)_0.10_ reaches 1.24 at 723 K via the trade-off between the degraded carrier mobility and the improved Seebeck coefficient, as well as the depressed lattice thermal conductivity. These results provide a reference for the implementation of entropy engineering in GeTe and other thermoelectric materials.

## 1. Introduction

On account of the rising energy costs and increasing global warming, the development of eco-friendly and sustainable energy technologies is a global challenge. Among them, thermoelectrics (TE) have been considered as one of the most compelling technologies due to the capability of direct conversion between heat and electricity, vibration-free operation, high reliability, and low environmental impact [1,2]. The energy conversion efficiency of the TE device is determined by the component materials’ dimensionless figure of merit, *zT* = *α*^2^*σT*/*κ*, where *T*, *α*, *σ* and *κ* denote the absolute temperature, Seebeck coefficient, electrical conductivity and thermal conductivity (including carrier component *κ*_e_ and lattice component *κ*_L_), respectively [3]. Aiming at decoupling the adversely interdependent TE parameters {*α*, *σ*, *κ*} and thus the high *zT*, the band engineering [4,5,6] and microstructure engineering [7,8,9,10,11] are implemented to enhance the power factor PF = *σα*^2^ and reduce the *κ*_L_, simultaneously. For TE materials undergoing phase transition, implementing phase engineering can also expand the phase favorable to thermoelectric and restrain the negative phase [12,13].

The configurational entropy Δ*S* characterizes the disorder of the system, and it is also an important parameter for developing high-performance TE materials [13]. Different from the traditional low-entropy alloys (LEA) with Δ*S* < 1 R, high-entropy alloys (HEA, Δ*S* > 1.5 R) consist of five or more major elements with a percentage of each atom between 5% and 35% [13]. High entropy alloys can provide a good way to improve thermoelectric properties by integrating the advantages of band, microstructure and phase engineering through multi-principal-element alloying [13,14,15,16,17,18,19,20,21]. So far, entropy engineering has been successfully applied to many TE systems, such as liquid-like materials [22,23,24], IV-VI compounds (including SnTe [20], PbSe [25], GeTe [13,26], and GeSe [27,28,29]), diamond-like compounds [19], Bi_2_Te_3_ [30], half-Heusler alloys [31], etc.

In multicomponent materials, Δ*S* has a profound effect on the TE parameters. First, the materials tend to form the high-symmetric cubic phase structure as long as the Δ*S* is high enough; meanwhile, high Δ*S* can reduce the structural phase transition temperature *T*_c_ [19,20,26]. The high crystal symmetry is generally inclined to possess large band degeneracy *N*_V_ and hence the large density-of-states’ effective mass m* [32]. Moreover, the high Δ*S* is conducive to increasing the solubility limit of specific elements, which in turn extends the phase space for performance optimization [20]. Therefore, higher *α* can be expected with higher Δ*S* value. Furthermore, the large differences of both atomic radius and mass among various components at the same sublattice site in multicomponent materials can result in a severe lattice distortion, which reduces the phonon velocities and enhances the phonon scattering simultaneously [19,20,33,34,35]. Hence, the *κ*_L_ can be reduced. However, it is noteworthy that although the multi-principal-element alloying can lower the *κ*_L_ towards the theoretical minimum as well as improve the *α* [13,16,19], the severe lattice distortion can also block the carrier transport, thus remarkably deteriorating the carrier mobility *μ*_H_ and *σ* that are bad for the *zT* advance. Consequently, the successful implementation of the entropy engineering presupposes that the fall in *μ*_H_ can be offset by the enhancement of *α* and the reduction of *κ*_L_ [20].

There are a number of strategies to solve the dilemma of *μ*_H_ reduction in multicomponent TE materials. For TE materials with carrier mean free path approaching to the Mott-Ioffe-Regel limit, multi-principal-element alloying does not further impair *μ*_H_, while only significantly reduces the *κ*_L_ and enhances *α* [17,19,20,30,31]. Unfortunately, this approach is only applicable to those TE systems with intrinsically ultralow *μ*_H_. Second, when designing high-entropy TE materials, the selected alloying element should have a small covalent radius difference in comparison with the host atoms in order to minimize the alloy scattering potential *E*_al_ and avoid the detrimental effect of high Δ*S* on *μ*_H_. Under this circumstance, only a few alloying elements are available for many TE systems, which also leads to a significant limitation in the implementation of entropy engineering. Last but not least, if the chosen alloying elements can enhance the m*, optimize the carrier concentration *n*_H_ and decline the *κ*_L_ as much as possible to compensate for the deterioration of *μ*_H_, the figure of merit can be surely enhanced [13,20]. Apparently, this is the easiest achievable design principle for implementing entropy engineering in thermoelectrics.

GeTe is a promising group IV-VI TE material for medium-temperature power generations [36]. Owing to the presence of a large number of Ge vacancies, pristine GeTe has a very high *n*_H_ (~10^21^ cm^−3^) at room temperature, giving rise to an extremely low *α* (~34 μV/K) and high *κ*_e_ (~5.65 W/mK) [37]. In addition, GeTe undergoes a phase transition from the rhombohedral (R-GeTe) to cubic structure (C-GeTe) in the temperature range of 600–700 K, relying on the *n*_H_ [12,13,17,38,39,40]. The phase transition of GeTe-based materials may induce high internal stresses and damage the GeTe-based devices under frequent thermal cycles or the material/electrode interfaces, hindering their practical application [40,41]. Up to now, carrier concentration optimization, suppressing rhombohedral-cubic phase transition, introducing multiscale microstructures and promoting multivalence band convergence are the prevalent strategies to improve the TE performance of GeTe-based alloys [5,26,38,42,43,44,45,46,47].

In this work, the Δ*S* of GeTe-based materials is regulated and the *zT* value is enhanced by progressive MnZnCdTe_3_ and Sb co-alloying, attesting to the efficacy of the design principle of rational selecting alloying elements in high-entropy thermoelectrics. Previous studies have demonstrated that sole Mn [38,47], Zn [44], or Cd [45,46] doping enabled the convergence of multivalence bands in GeTe and thereby increase m*. In the context of electron counting, replacing divalent Ge by isovalent Mn/Zn/Cd contributes no net *n*_H_ and therefore retains the *n*_H_ to the first order. To reduce the otherwise excessive *n*_H_ of GeTe, the introduction of trivalent Sb doping is a natural option [48]. In addition, MnZnCdTe_3_ and Sb co-alloying can increase the Δ*S* and lower *T*_c_ towards room temperature, which is beneficial to obtain large *N*_V_ and thus increase m* [26]. This offers the possibility to retain a decent PF in GeTe-based multicomponent materials through balancing the *μ*_H_ reduction and the *α* enhancement in the view of increased m* and optimized *n*_H_. Combined with the substantially diminished *κ*_L_ stemming from the severe lattice distortions and the micron-sized Zn_0.6_Cd_0.4_Te precipitations, the maximum *zT* ~1.24 at 723 K is attained in Ge_0.82_Sb_0.08_Te_0.90_(MnZnCdTe_3_)_0.10_. These results deepen the understanding of the rational selection of alloying elements in high-entropy TE materials.

## 2. Materials and Methods

### 2.1. Sample Synthesis and Preparation

Appropriate amounts of high-purity (99.999%) element chunks of Ge, Te, Mn, Zn, Cd and Sb were weighed according to the nominal composition of (GeTe)_1−*x*_(MnZnCdTe_3_)*_x_* (*x* = 0, 0.025, 0.050, 0.075, 0.100, and 0.125), as well as Ge_0.90−_*_y_*Sb*_y_*Te_0.90_(MnZnCdTe_3_)_0.10_ (*y* = 0.04, 0.06, 0.08, 0.10), sealed into quartz ampoules at 10^−3^ Pa, melted at 1373 K for 10 h and then quenched in cold water. The solidified ingots were ball milled (MSK-SFM-3, MTI Corporation) in a vacuum at 1200 rpm for 20 min to fine powders. Subsequently, the obtained fine powders were spark plasma sintered (SPS) into high-density cylinders with a diameter of 20 mm, with a thickness of 3 mm at 773 K for 5 min under the uniaxial pressure of 50 MPa.

### 2.2. Phase and Microstructure Characterization

The phase structures of all the GeTe-based samples were analyzed via the X-ray diffraction (XRD, CuKα, SmartLab, Rigaku^®^, Tokyo, Japan). The data analysis was performed via JADE 6.0 and the lattice parameters were calculated based on the Rietveld refinement method using the Topas 3.1 software. The phase transition temperature *T*_c_ from rhombohedral to cubic was inspected with a differential scanning calorimeter (DSC TAQ2000, New Castle, USA). The microstructures were investigated by scanning electron microscopy (SEM, Hitachi SU-70, Tokyo, Japan), and the chemical composition was examined by an energy dispersive spectrometer (EDS).

### 2.3. Transport Property Measurements

The measurements of the Seebeck coefficient *α* and electrical conductivity *σ* were conducted by a commercial ZEM-3 (Ulvac-Riko, Chigasaki, Japan) under a protective helium atmosphere. The measurement of thermal diffusivity *D* was performed on a Netzsch LFA 467 HT laser flash apparatus. The specific heat *C*_P_ was estimated by the Dulong-Petit law and the sample density *ρ* was determined via the Archimedes method. The thermal conductivity *κ* was then calculated as: *κ* = *ρC*_P_*D*. The carrier thermal conductivity *κ*_e_ was evaluated by the Wiedemann-Franz law, *κ*_e_ = *LσT*, where *L* is the Lorenz number and can be roughly expressed by $L=1.5+\;\exp\;\left[-\frac{\left|\alpha \right|}{116}\right]$ (where *L* is in 10^−8^ WΩK^−2^ and *α* in μV/K) [49]. The lattice thermal conductivity *κ***_L_** was calculated by subtracting *κ*_e_ from the total *κ*. The room temperature Hall coefficient *R*_H_ was measured on a PPMS system (Quantum Design^®^) with a magnetic field scanned between ±5 T. Subsequently, the carrier concentration *n*_H_ and Hall mobility *μ*_H_ were calculated according to the equation *n*_H_ = 1/e*R*_H_ and *μ*_H_ = *σR*_H_, respectively.

## 3. Results and Discussion

**Figure 1:**
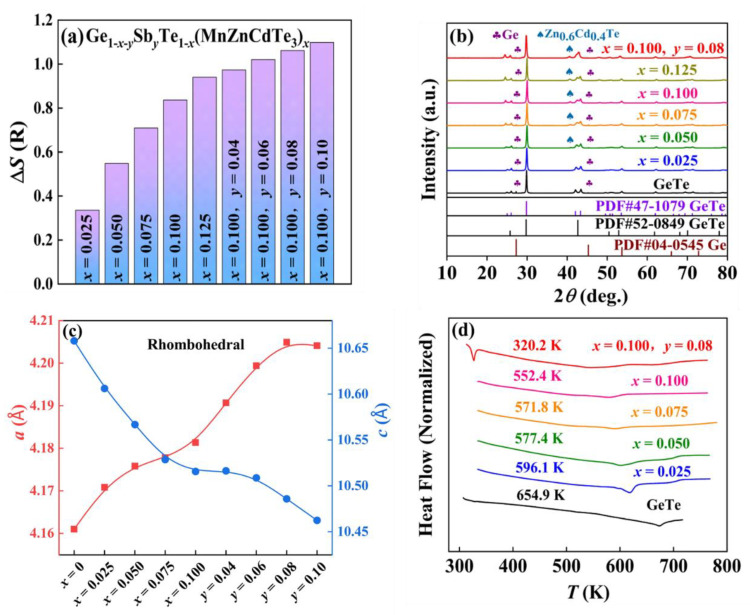
Room temperature (**a**) configurational entropy Δ*S*, (**b**) powder XRD patterns, (**c**) lattice parameter *a* and *c*, and (**d**) DSC curves and the obtained phase transition temperature *T*_c_ of GeTe-based alloys.

In fact, all the multicomponent GeTe-based alloys are inclined to form the high-symmetric cubic structures as long as the Δ*S* is sufficiently high [17]. Even if the entropy is not so high, boosting Δ*S* can still lead to the decrease in the structural transition temperature to some extent for GeTe-based alloys [13]. According to the Boltzmann theory, the configurational entropy can be defined as $\Delta S=-R\sum_{i=1}^{n}x_{i}\ln x_{i}$, where *n* is the number of alloying components in the solid solution, *x*_i_ is the mole fractions of the *i*th component, and *R* is the gas constant [14,15]. Obviously, Δ*S* increases from 0 *R* for binary GeTe to 0.84 *R* for (GeTe)_0.90_(MnZnCdTe_3_)_0.10_ and then to 1.06 *R* with Sb co-doping in Ge_0.82_Sb_0.08_Te_0.90_(MnZnCdTe_3_)_0.10_, reaching the medium-entropy region (Figure 1a). As observed from the room-temperature powder X-ray diffraction (XRD) pattern (Figure 1b), the double peaks in the 2*θ* ranges of both 23°–27° and 41°–45° indicate the rhombohedral structure in pristine GeTe [12,50]. As Δ*S* rises, the double peaks gradually converge into a single peak, demonstrating that the room-temperature crystal structure gradually changes from R-GeTe to C-GeTe [13,26]. The lattice parameters *a* and *c* are calculated based on the powder XRD diffraction patterns and the results are plotted in Figure 1c. Typically, the lattice parameter *a* is 4.161 Å and *c* is 10.658 Å for pristine GeTe. When Δ*S* ascends to 0.84 *R*, *a* ascends to 4.181 Å and *c* descends to 10.516 Å for the (GeTe)_0.90_(MnZnCdTe_3_)_0.10_ sample. Further increasing Δ*S* to 1.06 *R*, *a* rises to 4.205 Å and *c* drops to 10.486 Å for the Ge_0.82_Sb_0.08_Te_0.90_(MnZnCdTe_3_)_0.10_ sample, demonstrating the promotion of the rhombohedral-cubic phase transition with the increasing Δ*S*.

Differential scanning calorimetry (DSC) analysis is carried out to further detect the variation of *T*_c_ upon progressive MnZnCdTe_3_ and Sb co-alloying. As depicted in Figure 1d, the *T*_c_ of pristine GeTe is 655 K, and gradually falls to 552 K for (GeTe)_0.90_(MnZnCdTe_3_)_0.10_ with the increasing Δ*S*. When further boosting Δ*S*, the *T*_c_ of Ge_0.82_Sb_0.08_Te_0.90_(MnZnCdTe_3_)_0.10_ sample declines to 320 K, which is close to the room temperature. This indicates that the increase in Δ*S* can observably depress the *T*_c_ and is beneficial to obtain large *N*_V_ and thus high m* [13,26], which will be discussed later.

**Figure 2:**
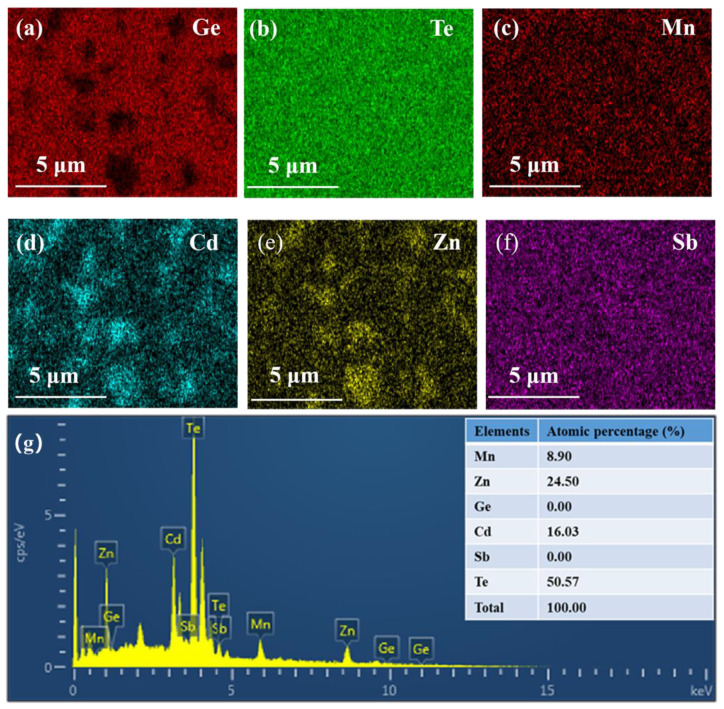
The scanning electron microscopy (SEM) and the X-ray energy dispersive spectrum (EDS) elemental spot scanning of the polished Ge_0.82_Sb_0.08_Te_0.90_(MnZnCdTe_3_)_0.10_. The element mapping of (**a**) Ge, (**b**) Te, (**c**) Mn, (**d**) Cd, (**e**) Zn, (**f**) Sb, and (**g**) the X-ray energy dispersive spectrum analysis, indicating the micron-sized Zn_0.6_Cd_0.4_Te precipitations are existed.

In addition to stabilizing the high-symmetry C-GeTe phase, the high Δ*S* also inclines to extend the solubility limits of specific elements [20]. Typically, there are a large number of Ge precipitations as well as Ge vacancies in pristine GeTe [37,51,52]. Two weak diffraction peaks of Ge precipitation can be observed at 27.0°–27.5° and 45.0°–45.5° on the XRD pattern of binary GeTe, as marked in Figure 1b. Interestingly, the diffraction peaks of Ge secondary phases gradually diminish and eventually disappear with increasing Δ*S*. For the multicomponent system, the Gibbs free energy of mixing Δ*G* is determined by ΔG=ΔH−TΔS, where Δ*H* is the enthalpy of mixing. Obviously, if the Δ*H* keeps constant, a higher Δ*S* will give rise to a lower Δ*G* [20]. Under this scenario, the high Δ*S* may promote the Ge precipitations to be dissolved into the matrix, which in turn reduces the excessive Ge vacancies and thus mildly decreases the *n*_H_. Furthermore, Zn_0.6_Cd_0.4_Te precipitations are observed when the MnZnCdTe_3_ alloying content *x* reaches 5%, indicating the low solubility limit of MnZnCdTe_3_ in GeTe. The SEM-EDS images further confirm that the dark grey regions of 0.1–2.0 μm is Zn_0.6_Cd_0.4_Te, which are embedded in the host matrix of GeTe-based multicomponent alloys (Figure 2). These micron-sized Zn_0.6_Cd_0.4_Te secondary phases are favorable for the enhancement of low-frequency phonons scattering, which is beneficial to obtain the low *κ*_L_. It is consistent with our aim of reducing *κ*_L_ by multi-component alloying. However, the low solubility limit of MnZnCdTe_3_ is not conducive to promoting band convergence and suppressing the rhombohedral-cubic phase transition, which in turn restrains the improvement of m* and PF.

**Figure 3:**
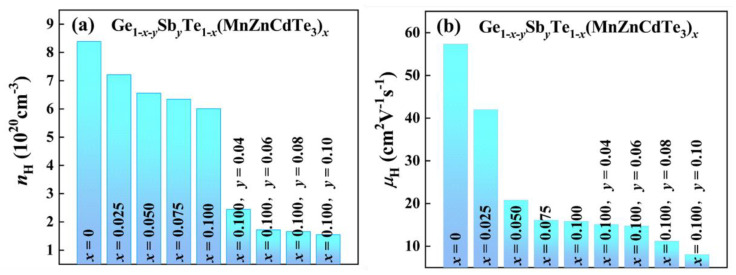
The room temperature (**a**) carrier concentration *n*_H_, and (**b**) carrier mobility *μ*_H_ of Ge_1−*x*−*y*_Sb*_y_*Te_1−*x*_(MnZnCdTe_3_)*_x_* samples.

In order to analyze the mechanism underlining the variation of electrical transport properties, the room temperature *n*_H_ and *μ*_H_ for all the Ge_1−*x*−*y*_Sb*_y_*Te_1−*x*_(MnZnCdTe_3_)*_x_* samples are measured. As presented in Figure 3a, the *n*_H_ falls mildly with increasing MnZnCdTe_3_ alloying content. For example, the room temperature *n*_H_ decreases from 8.4 × 10^20^ cm^−3^ for GeTe to 6.0 × 10^20^ cm^−3^ for (GeTe)_0.90_(MnZnCdTe_3_)_0.10_. Theoretically, single element doping with the isovalent Zn, Cd or Mn at Ge site will not have a significant effect on *n*_H_ [38,44,45,46,47]. The previous literature has demonstrated that isoelectronic Mn alloying can even promote the formation of Ge vacancy and thus boost the *n*_H_ [38,47]. In the present work, the anomalous decrease in *n*_H_ after Mn-Zn-Cd co-alloying may be ascribed to the re-dissolution of Ge precipitations into the GeTe matrix via a high entropy effect, which is in line with the XRD results. Subsequently, the donor doping of trivalent Sb further reduces the *n*_H_ substantially to 1.7 × 10^20^ cm^−3^ for Ge_0.82_Sb_0.08_Te_0.90_(MnZnCdTe_3_)_0.10_, approaching to the optimal carrier concentration of *n*_opt_ (~2 × 10^20^ cm^−3^) for rhombohedral GeTe-based alloys [48,52,53].

Severe lattice distortion induced by multi-principal-element alloying can impede the carrier transport and hence degrade the *μ*_H_ [13,20]. As illustrated in Figure 3b, the MnZnCdTe_3_ alloying dramatically deteriorates the *μ*_H_ of GeTe. Typically, the room temperature *μ*_H_ drastically reduces from 57.3 cm^2^V^−1^s^−1^ for pristine GeTe to 20.8 cm^2^V^−1^s^−1^ for (GeTe)_0.95_(MnZnCdTe_3_)_0.05_ and further to 15.8 cm^2^V^−1^s^−1^ for (GeTe)_0.90_(MnZnCdTe_3_)_0.10_. Moreover, Mn alloying will flatten the valence band edge and largely increase the band effective mass *m*_b_* of GeTe, leading to a remarkable deterioration in *μ*_H_. For instance, Zheng et al. [47] reported that the room temperature m* increased quickly from 1.44 *m*_0_ for binary GeTe to 6.15 *m*_0_ for Ge_0.85_Mn_0.15_Te, corresponding to a catastrophic reduction of room temperature *μ*_H_ from 54.17 cm^2^V^−1^s^−1^ to 4.41 cm^2^V^−1^s^−1^. Similarly, Liu et al. found that the room temperature m* for pristine GeTe was only 1.6 *m*_0_, which was then markedly enhanced to 36.7 *m*_0_ after 30 at% Mn alloying. Meanwhile, the room temperature *μ*_H_ diminished rapidly from 95.3 cm^2^V^−1^s^−1^ to 0.5 cm^2^V^−1^s^−1^ [38]. In view of the deterioration of *μ*_H_ approaching to the Mott-Ioffe-Regel limit upon MnZnCdTe_3_ alloying, further Sb alloying does not impair the *μ*_H_, but Sb alloying can concurrently suppress the rhombohedral-cubic phase transition, optimize the *n*_H_ and reduce the *κ*_L_ [26,31,48].

**Figure 4:**
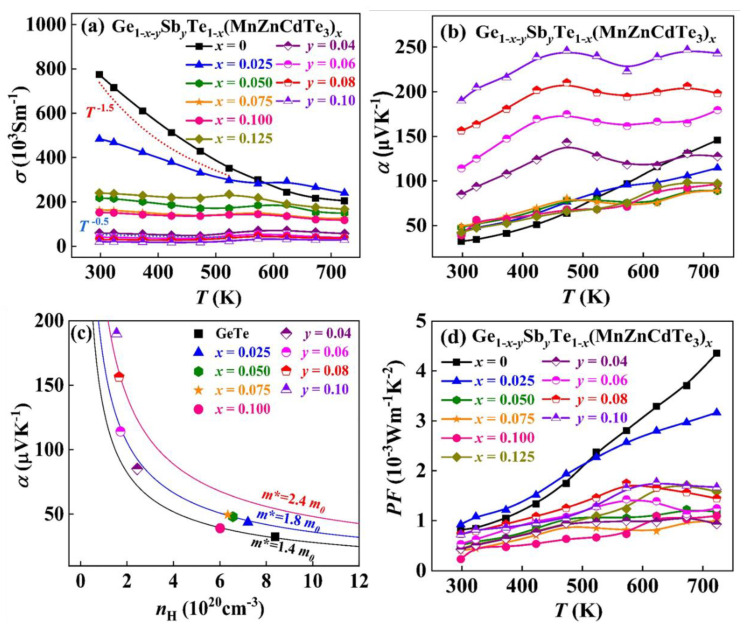
Temperature dependence of (**a**) electrical conductivity *σ*, (**b**) Seebeck coefficient *α*, and (**d**) power factor PF for (GeTe)_1−*x*_(MnZnCdTe_3_)*_x_* and Ge_0.82−*y*_Sb*_y_*Te_0.90_(MnZnCdTe_3_)_0.10_ samples. (**c**) Room temperature Seebeck coefficient *α* as a function of carrier concentration *n*_H_ for Ge_1−*x*−*y*_Sb*_y_*Te_1−*x*_(MnZnCdTe_3_)*_x_* samples.

In general, the high Δ*S* is adverse to high *σ* [13,20]. Figure 4a depicts the temperature dependence of *σ* for all the GeTe-based samples. The *σ* of pristine GeTe is extremely high because of the high *n*_H_ and *μ*_H_. After MnZnCdTe_3_ alloying, the *σ* decreases dramatically due to the simultaneous reduction of *n*_H_ and *μ*_H_, which originates from the dissolution of Ge precipitations, band flattening and severe lattice distortion. Typically, the room temperature *σ* decreases from 774 × 10^3^ Sm^−1^ for binary GeTe to 152 × 10^3^ Sm^−1^ for (GeTe)_0.90_(MnZnCdTe_3_)_0.10_. In light of the *μ*_H_ approaching to the Mott-Ioffe-Regel limit, further Sb alloying reduces the *n*_H_ but has a weak impact on carrier scattering, thereby bringing out a further decreased *σ*. Therefore, the room temperature *σ* of the Ge_0.82_Sb_0.08_Te_0.90_(MnZnCdTe_3_)_0.10_ sample decreases to 30 × 10^3^ Sm^−1^. In addition, the *σ* of pristine GeTe exhibits a temperature dependence of *T*^−1.48^, indicating that the acoustic phonon scattering (*σ* ~*T*^−1.5^) is the dominant scattering mechanism in this system. The power law exponent of the *σ* gradually decreases with increasing Δ*S* because of the enhanced point defect scattering. Thus, the temperature dependence of *T*^−0.4^ is obtained in Ge_0.82_Sb_0.08_Te_0.90_(MnZnCdTe_3_)_0.10_, which is consistent with the alloy scattering mechanism (*σ* ~*T^−^*^0.5^) [54].

The progressive alloying of MnZnCdTe_3_ and Sb increases Δ*S* and thus suppresses *T*_c_ near room temperature, which in turn enhances the m* and hence *α* [13,26]. As exhibited in Figure 4b, the *α* boosts mildly near room temperature upon MnZnCdTe_3_ alloying while increases substantially in the whole temperature range after Sb doping. For example, the room temperature *α* sharply increases from 32.5 μVK^−1^ for binary GeTe to 156.4 μVK^−1^ for Ge_0.82_Sb_0.08_Te_0.90_(MnZnCdTe_3_)_0.10_.

To gain insight into the beneficial effect of band structure variation on *α*, the Pisarenko plots are calculated according to the single parabolic band (SPB) model [55]. As demonstrated in Figure 4c, the m* rises from 1.4 *m*_0_ for the GeTe sample to 1.8 *m*_0_ for the MnZnCdTe_3_ alloyed sample and finally increases to 2.4 *m*_0_ for the Ge_0.82_Sb_0.08_Te_0.90_(MnZnCdTe_3_)_0.10_ sample. On the one hand, the increase in Δ*S* lowers the *T*_c_ of GeTe, resulting in large *N*_V_ and thus m*. On the other hand, the co-alloying of Mn, Zn and Cd is conductive to promoting the multivalence band convergence and valence band flattening, which further enhances m* [38,44,45,47]. Notably, the low solubility limit of MnZnCdTe_3_ in GeTe is detrimental to band structure modulation. Besides the increase in m*, both the suppression of Ge vacancies via high entropy effect and the trivalent Sb donor doping contribute to a large reduction of *n*_H_, giving rise to a huge enhancement of *α*. The increased m* and optimized *n*_H_ partially compensate for the deterioration of *μ*_H_, thus attaining a decent power factor PF in the whole temperature region (Figure 4d) as well as a maximum PF of 1.75 × 10^−3^ Wm^−1^K^−2^ for Ge_0.82_Sb_0.08_Te_0.90_(MnZnCdTe_3_)_0.10_.

**Figure 5:**
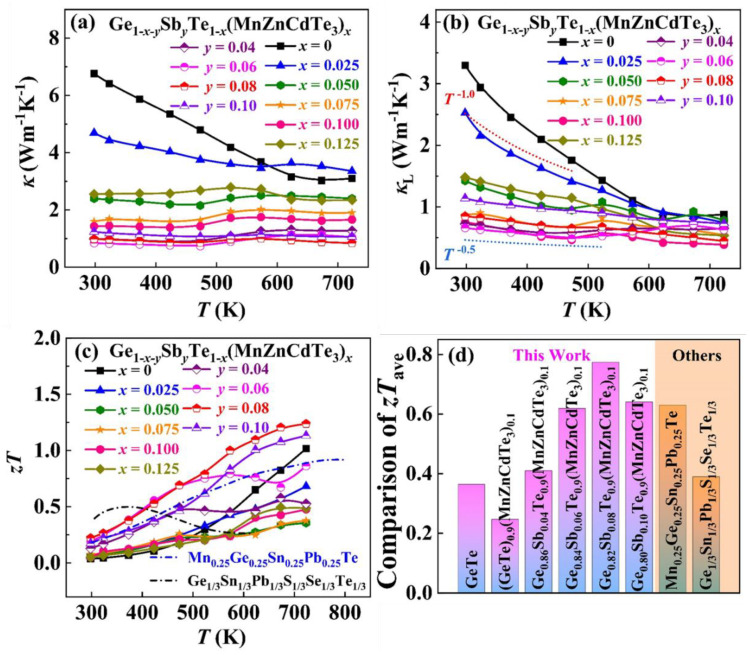
Temperature dependences of (**a**) total thermal conductivity *κ*, (**b**) lattice thermal conductivity *κ*_L_, and (**c**) *zT* of (GeTe)_1−*x*_(MnZnCdTe_3_)*_x_* and Ge_0.82−*y*_Sb*_y_*Te_0.90_(MnZnCdTe_3_)_0.10_ samples. (**d**) The average *zT*_ave_ values between 298–723 K for our GeTe-based alloys.

The high *κ* (8 Wm^−1^K^−1^ at 300 K) limits the TE performance of *p*-type binary GeTe [36]. Therefore, it is necessary to reduce the otherwise too high *κ* in GeTe-based alloys. The severe lattice distortion induced by multi-principal-element alloying affords a natural option [15,19,20]. The temperature-dependent *κ* for all the GeTe-based alloys is plotted in Figure 5a. As expected, the *κ* of multi-principal-elements alloys are much lower than the pristine GeTe, because of the concurrent reduction of both *κ*_e_ (due to the declined *σ*) and *κ*_L_. The room temperature *κ* substantially drops from 6.76 Wm^−1^K^−1^ for binary GeTe to 1.43 Wm^−1^K^−1^ for (GeTe)_0.90_(MnZnCdTe_3_)_0.10_ and then to 0.98 Wm^−1^K^−1^ for Ge_0.82_Sb_0.08_Te_0.90_(MnZnCdTe_3_)_0.10_.

The *κ*_L_ of all the samples is presented in Figure 5b. In view of the severe lattice distortions induced by large size and mass differences among various components at the same sublattice site, the multi-principal-element alloying can effectively minimize the *κ*_L_ of GeTe [13,20]. Moreover, the micron-sized Zn_0.6_Cd_0.4_Te secondary phases were caused by the low solubility limit of MnZnCdTe_3_ in GeTe (Figure 2) that is conducive to scattering low-frequency phonons. The synergy of aforesaid two factors greatly diminish the *κ*_L_ throughout the measured temperature region. Therefore, the room-temperature *κ*_L_ declines from 3.30 Wm^−1^K^−1^ for binary GeTe to 0.75 Wm^−1^K^−1^ for (GeTe)_0.90_(MnZnCdTe_3_)_0.10_ and further falls to 0.66 Wm^−1^K^−1^ for Ge_0.82_Sb_0.08_Te_0.90_(MnZnCdTe_3_)_0.10_. In particular, the minimum *κ*_L_ of 0.45 Wm^−1^K^−1^ at 723 K is achieved in Ge_0.82_Sb_0.08_Te_0.90_(MnZnCdTe_3_)_0.10_, approaching the theoretical minimum *κ*_L_ (~0.3 Wm^−1^K^−1^) of GeTe [17]. Moreover, the power law exponent of *κ*_L_ decreases from −1.25 for binary GeTe to −0.45 for Ge_0.82_Sb_0.08_Te_0.90_(MnZnCdTe_3_)_0.10_, indicating that the dominant phonon scattering alters from acoustic phonon scattering (*κ*_L_ ~*T*^−1.0^) to point defect scattering (*κ*_L_ ~*T*^−0.5^) with increasing alloying species and content [20].

The trade-off between the enhanced *α*, the depressed *κ*_L_ and the deteriorated *μ*_H_ determines the variation of *zT* values with increasing Δ*S* (Figure 5c). Notably, the maximum *zT* of 1.24 at 723 K is attained in the Ge_0.82_Sb_0.08_Te_0.90_(MnZnCdTe_3_)_0.10_ sample through entropy engineering, about 22% increment over the pristine GeTe. The peak *zT* in this study is much higher than those of the high-entropy Ge_1/3_Sn_1/3_Pb_1/3_Te_1/3_Se_1/3_S_1/3_ (~0.51) [18] and Ge_0.25_Sn_0.25_Pb_0.25_Mn_0.25_Te alloys (~0.92) [19] without a rational selection of alloying species. In addition, it is noteworthy that the average *zT*_ave_ value between 298 K and 723 K is 0.77, which is 113% higher than that of binary GeTe (Figure 5d). In particular, the *zT*_ave_ in this work is also much higher than those of high-entropy Ge_1/3_Sn_1/3_Pb_1/3_Te_1/3_Se_1/3_S_1/3_ (~0.39) and Ge_0.25_Sn_0.25_Pb_0.25_Mn_0.25_Te alloys (~0.63). These results demonstrate the validity of the screening principle for alloying species in high-entropy TE materials.

## 4. Conclusions

In summary, we demonstrate the power of entropy engineering on balancing the power factor and lattice thermal conductivity of GeTe in line with the rationally screening alloying species. To establish the structure-property correlation in GeTe-based multicomponent materials, entropy engineering is adopted to design the GeTe-based alloys with progressive MnZnCdTe_3_ and Sb co-alloying based on the nominal composition of (Ge_1−*x*−*y*_Sb*_y_*Te_1−*x*_)(MnZnCdTe_3_)*_x_*. Multi-principal-element alloying can regulate the configurational entropy and thus improve the thermoelectric properties of the GeTe-based materials. The phase structures, microstructures and thermoelectric properties of the obtained alloys are systematically studied. The high configurational entropy reduces the rhombohedral-cubic phase transition temperature to near room temperature and Mn-Zn-Cd co-alloying promotes the multivalent bands’ convergence, which are both conducive to obtaining a high density-of-state effective mass. Meanwhile, the synergy of the decreased Ge vacancies content via the dissolution of Ge precipitations and trivalent Sb donor doping reduces the excessive carrier concentration of GeTe-based alloys. The optimized carrier concentration and the increase in the density-of-state effective mass improve the Seebeck coefficient substantially, which counteracts the decrease in electrical conductivity. Moreover, the severe lattice distortions and micron-sized Zn_0.6_Cd_0.4_Te second phases result in a significant reduction of lattice thermal conductivity. As a result, the maximum *zT* value of 1.24 at 723 K is attained in Ge_0.82_Sb_0.08_Te_0.90_(MnZnCdTe_3_)_0.10_. These results not only prove the validity of rational screening principle for alloying species in high-entropy thermoelectric materials, but also trigger profound thoughts on the application of emerging entropy engineering in the field of thermoelectric materials.

## Data Availability

The data presented in this study are available on request from the corresponding author.
